# Three Cases of Biliary Disease Presenting With Chest Pain: Diagnostic Pitfalls and the Impact of Cognitive Biases in Noncardiac Chest Pain

**DOI:** 10.7759/cureus.98993

**Published:** 2025-12-11

**Authors:** Atsuo Maeda, Hikaru Kawakita, Tomoko Yashima, Go Haraguchi, Masahito Uchino

**Affiliations:** 1 Department of Emergency and Critical Care Medicine, Kawakita General Hospital, Tokyo, JPN

**Keywords:** acute cholecystitis, anchoring bias, availability bias, biliary disease, cholangitis, cognitive bias, diagnostic delay, emergency medicine, framing bias, noncardiac chest pain

## Abstract

Chest pain is a frequent chief complaint among patients visiting the emergency department, and those with acute coronary syndrome (ACS) are usually prioritized during initial evaluation. However, biliary diseases occasionally mimic cardiac chest pain, leading to diagnostic delays. We describe three patients who presented with chest or epigastric pain and were initially suspected of having ACS. In each case, cardiac biomarkers and electrocardiograms were nondiagnostic, whereas liver or biliary enzyme abnormalities and imaging studies revealed acute cholecystitis or cholangitis. All patients underwent appropriate surgical or endoscopic intervention and achieved complete recovery. These cases highlight that biliary disease can present as noncardiac chest pain because of shared visceral afferent pathways. Clinicians should systematically reassess for abdominal sources after ACS is excluded, particularly when liver enzyme levels are elevated or Murphy’s sign is positive. Awareness of cognitive biases, including anchoring, availability, and framing, is essential for avoiding diagnostic delays in emergency settings.

## Introduction

Chest pain is a common chief complaint in the emergency department (ED), and clinical evaluation often prioritizes the exclusion of acute coronary syndrome (ACS) [1,2]. In many cases, the evaluation of noncardiac causes begins only after ACS is ruled out [3]. Only about 5.1% ED patients presenting with chest pain are ultimately diagnosed with ACS, and more than half have a noncardiac cause [2]. Among these noncardiac etiologies, gastrointestinal conditions are common, and biliary disease, although less frequent, has been reported to mimic ACS and even lead to initial misdiagnosis [4]. Cognitive biases are also recognized as common contributors to diagnostic error and delay in the ED, and framing and anchoring biases are particularly relevant when atypical presentations, such as biliary disease, mimic ACS [5]. Here, we report three cases in which patients were initially suspected of having ACS but were ultimately diagnosed with biliary disease. We discuss the importance of differential diagnosis of noncardiac chest pain and the influence of cognitive biases on clinical decision-making.

## Case presentation

Case 1

**Figure 1:**
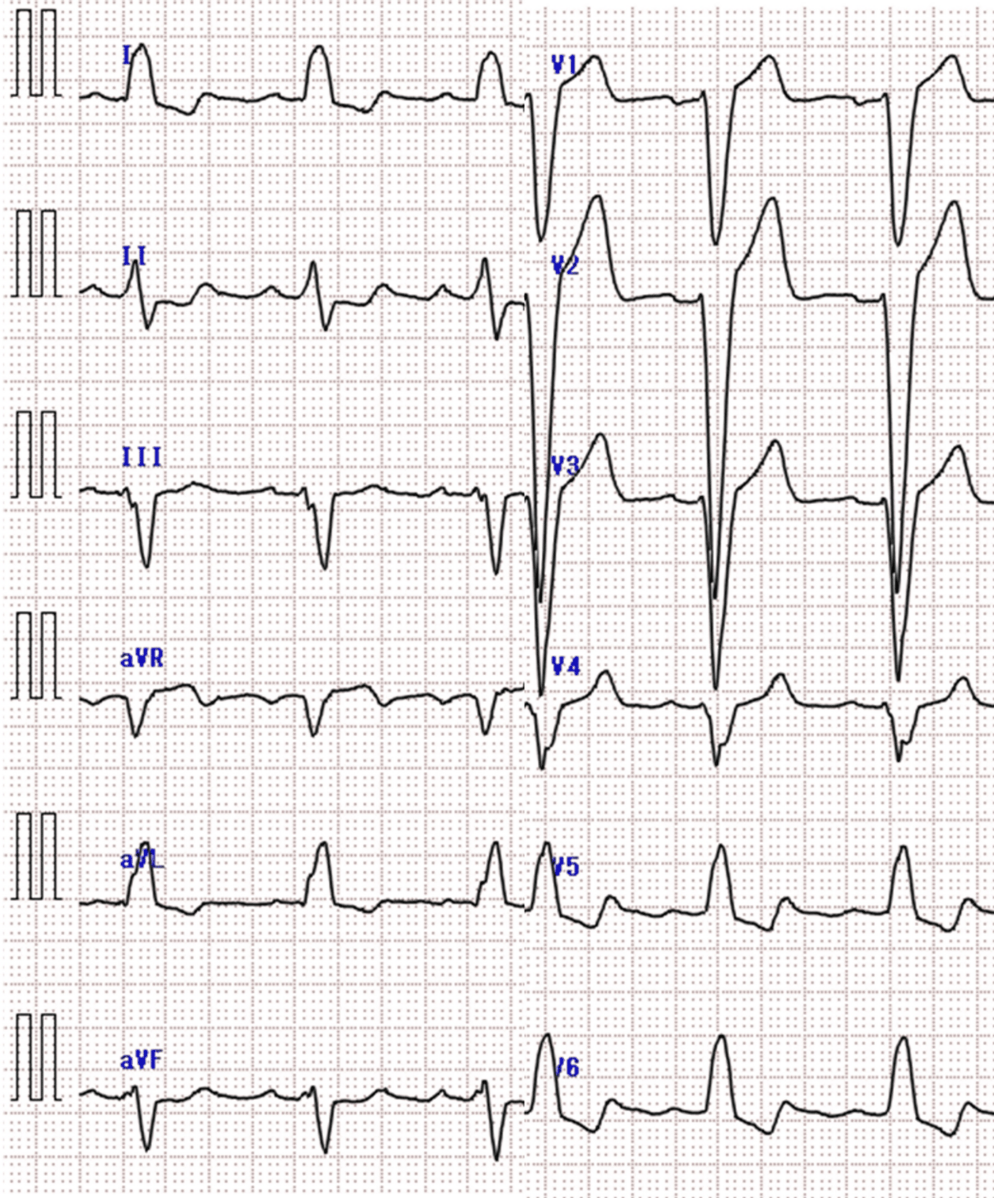
Electrocardiographic findings in Case 1 Electrocardiogram showing T-wave inversion in lead I without ST elevation

A 50-year-old man with no significant medical history presented with a sudden onset of intermittent, stinging chest pain lasting for two hours. By the time he arrived at the hospital, the pain had subsided, and his vital signs were stable. A 12-lead electrocardiography (ECG) revealed a left bundle branch block, which was also present on a previous ECG (Figure 1). The Sgarbossa criteria were considered but not met [6]. Cardiac biomarkers were within normal limits, whereas liver enzymes were mildly elevated (Table 1). Because the chest pain had resolved and ACS was deemed unlikely, the patient was discharged.

**Table 1:** Laboratory findings in Case 1 CRP: C-reactive protien; AST: aspartate aminotransferase; ALT: alanine transaminase; T-bil: total bilirubin; γ‐GTP: gamma-glutamyl transferase; ALP: alkaline phosphatase; CK: creatine kinase; Tn-1: troponin I

Parameters	Patient Values at First Presentation	Patients Values at Second Presentation	Units	Reference Ranges
WBC	7300	10200	/μL	3500-8500
CRP	0.05	0.06	mg/dl	0-0.29
AST	74	316	IU/L	7-38
ALT	56	185	IU/L	4-36
T-bil	0.92	1.27	mg/dl	0.2-1.2
γ‐GTP	89	240	IU/L	9-40
ALP	78	108	IU/L	38-113
CK	162	201	IU/L	60-290
Tn-I	10	10	pg/ml	0-26.2

He was instructed to return promptly if chest or abdominal pain recurred, and an outpatient follow-up was arranged. At that time, abdominal imaging was not performed because ACS had been excluded, the pain had resolved, and the mildly abnormal liver function tests were not initially regarded as clinically significant. The medical team was reassured by excluding ACS, but further diagnostic evaluation was not pursued.

The following day, he experienced recurrent chest pain and was transported via emergency medical services to the Tokyo Coronary Care Unit (CCU) Network with suspected ACS. He complained of chest pain and nausea. On arrival, the patient was alert (Glasgow Coma Scale E4V5M6). His vital signs were stable: respiratory rate, 18 breaths per minute; pulse, 72 beats per minute; blood pressure, 139/85 mmHg; oxygen saturation, 97% on room air; and body temperature, 36.8°C. Pupils were equal at 3 mm bilaterally and reactive to light.

**Figure 2:**
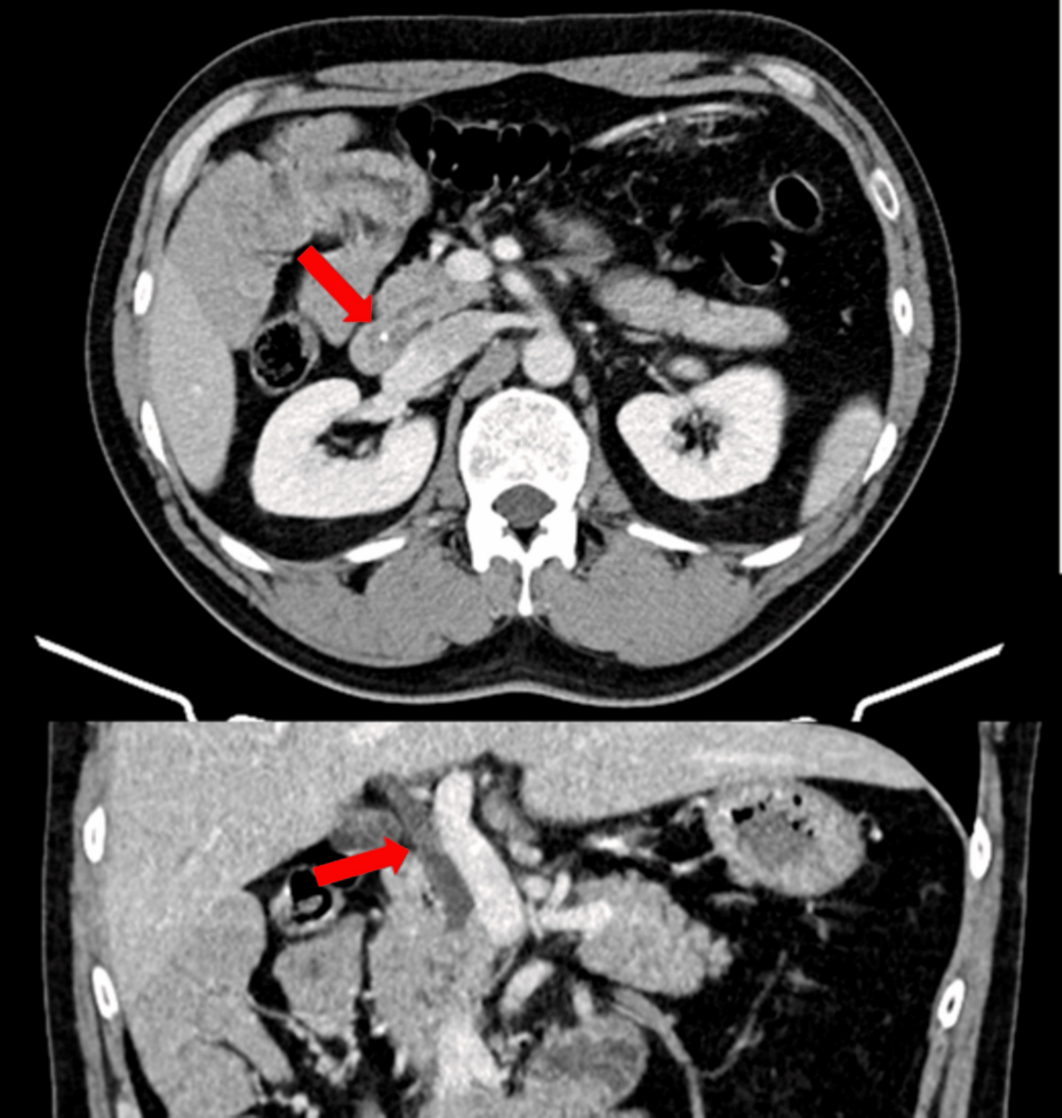
CT findings in Case 1 Abdominal CT showing gallbladder enlargement and wall thickening, consistent with acute calculous cholecystitis (red arrow).

Physical examination revealed no conjunctival pallor or scleral icterus. Breath sounds were clear bilaterally, and no cardiac murmurs were heard. He reported spontaneous pain extending from the anterior chest to the epigastric region, but there was no tenderness on palpation. The abdomen was flat and soft, with normal bowel sounds, and no lower extremity edema was observed. The ECG findings were unchanged from the previous day. Cardiac markers remained within normal limits; however, liver enzymes had worsened (Table 1). A contrast-enhanced abdominal CT scan was performed to evaluate the liver function abnormalities, revealing dilation of the common bile duct and the presence of common bile duct stones (Figure 2).

According to the Tokyo Guidelines 2018 (TG18) diagnostic criteria, the presence of systemic inflammation (mild leukocytosis), cholestasis (elevated biliary enzymes), and imaging findings of choledocholithiasis fulfilled the diagnosis of mild acute cholangitis. Guideline-directed intravenous cefmetazole therapy was initiated the same day, and endoscopic retrograde cholangiopancreatography (ERCP) was performed electively on day 2. Bile culture grew *Peptoniphilus asaccharolyticus*, which was susceptible to cefmetazole. Antibiotic therapy was continued for seven days, and the patient was discharged without symptoms on day 8.

Case 2

A 50-year-old man with a history of ST-elevation myocardial infarction (STEMI) and percutaneous coronary intervention one month ago presented with sudden epigastric pain resembling his previous cardiac event. He was transported to our hospital via the Tokyo CCU Network because of the suspicion of ACS recurrence.

On arrival, the patient was alert (Glasgow Coma Scale E4V5M6). His vital signs were as follows: respiratory rate, 14 breaths per minute; pulse, 93 beats per minute; blood pressure, 138/94 mmHg; oxygen saturation, 97% on room air; and body temperature, 36.7°C. Pupils were equal at 3 mm bilaterally and reactive to light.

**Figure 3:**
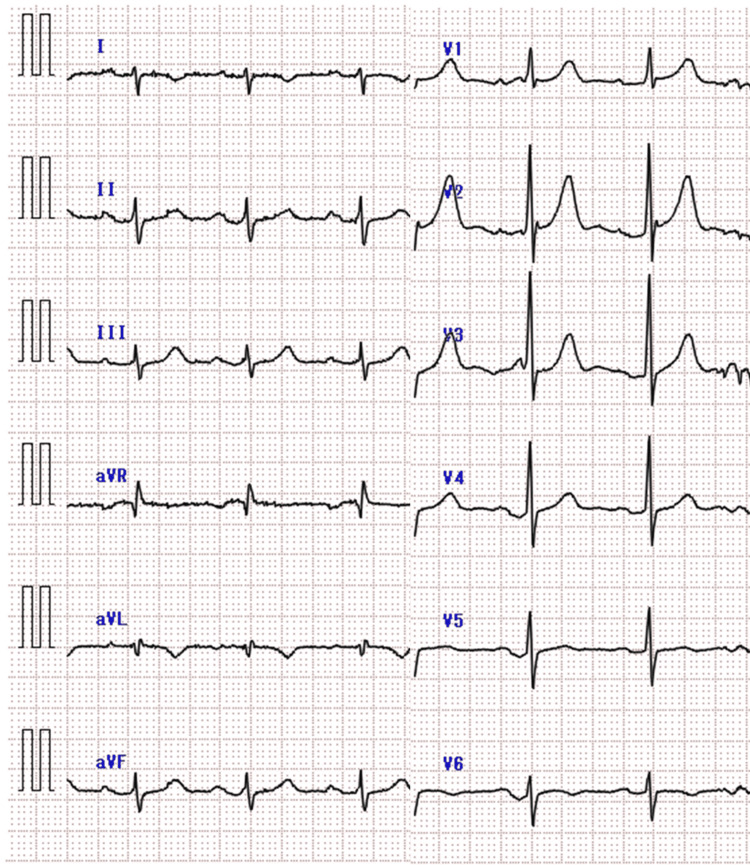
Electrocardiographic findings in Case 2 Electrocardiogram showing T-wave inversion in lead I without ST elevation

Physical examination revealed no conjunctival pallor or scleral icterus. Breath sounds were clear bilaterally, and no cardiac murmurs were heard. The patient reported spontaneous pain extending from the anterior chest to the epigastric region but had no tenderness on palpation. The abdomen was flat and soft, with normal bowel sounds, and no lower extremity edema was noted. ECG revealed T-wave inversion in leads I and aVL without ST elevation (Figure 3). Cardiac markers remained within normal limits; however, alkaline phosphatase (ALP), γ-GTP, and amylase levels were slightly elevated. The white blood cell count was elevated (Table 2).

**Table 2:** Laboratory findings in Case 2 CRP: C-reactive protien; AST: aspartate aminotransferase; ALT: alanine transaminase; T-bil: total bilirubin; γ‐GTP: gamma-glutamyl transferase; ALP: alkaline phosphatase; CK: creatine kinase; Tn-1: troponin I

Parameters	Patient Values	Units	Reference Ranges
WBC	19700	/μL	3500-8500
CRP	0.13	mg/dl	0-0.29
AST	28	IU/L	7-38
ALT	31	IU/L	4-36
T-bil	0.42	mg/dl	0.2-1.2
γ‐GTP	85	IU/L	9-40
ALP	148	IU/L	38-113
CK	56	IU/L	60-290
Tn-I	20.2	pg/ml	0-26.2

**Figure 4:**
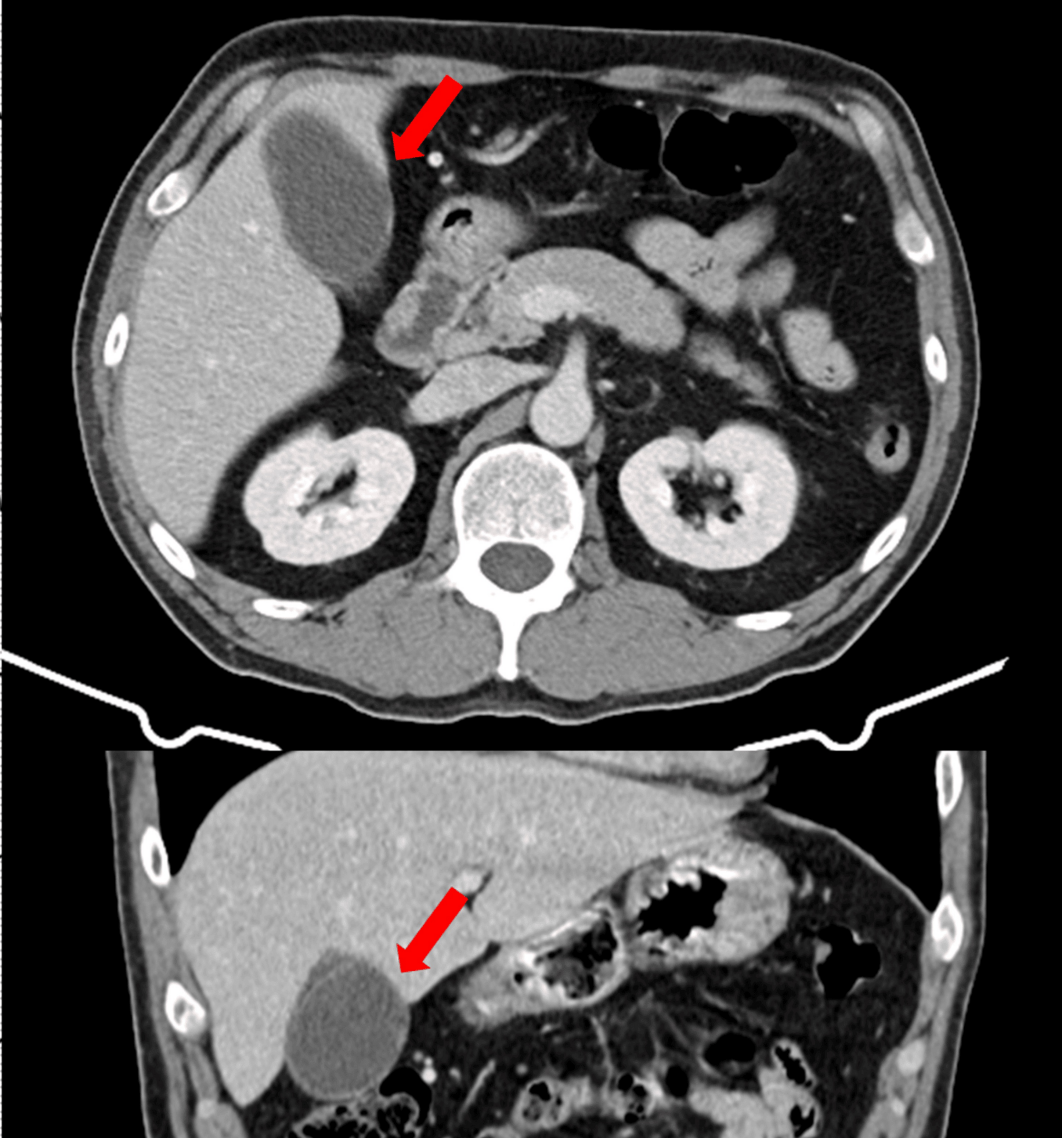
CT findings in Case 2 Abdominal CT showing gallbladder enlargement and wall thickening, consistent with acute calculous cholecystitis (red arrow).

Because ACS and aortic dissection were initially considered, a contrast-enhanced chest-abdominal CT scan was performed. Although acute cholecystitis was not suspected at first, an ultrasound was not performed. The CT revealed gallbladder enlargement and wall thickening (Figure 4).

A repeat physical examination confirmed a positive Murphy’s sign. According to the TG18 diagnostic criteria, the patient fulfilled the requirements for acute cholecystitis, showing local signs (positive Murphy’s sign), systemic inflammation, and imaging findings. Acute cholecystitis was considered the most likely diagnosis based on the clinical presentation. The patient had undergone percutaneous coronary intervention (PCI) at another hospital one month earlier. At our facility, serial troponin measurements were not performed, and no baseline ECG was available for comparison. Because comparison with previous examinations was not possible and stent-related complications could not be completely ruled out, the patient was transferred to the hospital where PCI had been performed. Further management of acute cholecystitis and reevaluation for possible cardiac disease were requested. At the receiving hospital, a laparoscopic cholecystectomy was performed, confirming the diagnosis of acute cholecystitis.

Case 3

**Figure 5:**
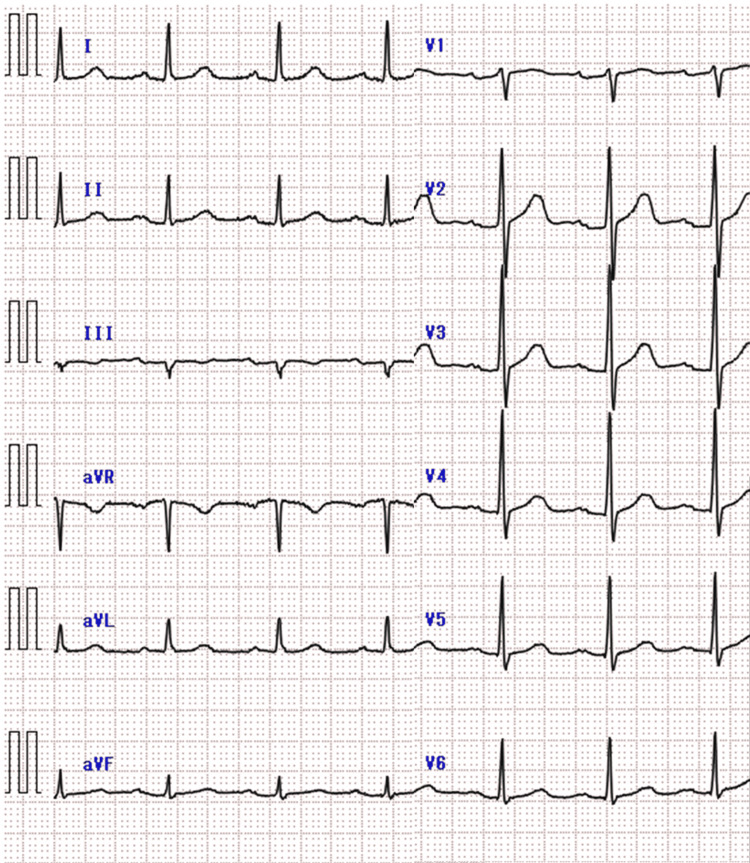
Electrocardiographic findings in Case 3 Electrocardiogram showing no significant abnormalities

A 77-year-old man with a history of hypertension and dyslipidemia presented with anterior chest and epigastric pain at rest. He was referred to our ED from a local clinic because of suspected angina. On arrival, the patient reported pain extending from the left anterior chest to the epigastrium. The patient was alert (Glasgow Coma Scale E4V5M6). His vital signs were as follows: respiratory rate, 16 breaths per minute; pulse, 79 beats per minute; blood pressure, 149/85 mmHg; oxygen saturation, 96% on room air; and body temperature, 36.9℃. Pupils were equal at 3 mm bilaterally and reactive to light. Physical examination revealed no conjunctival pallor, scleral icterus, or cervical lymphadenopathy. Breath sounds were clear bilaterally, and no cardiac murmurs were heard. The patient reported spontaneous pain extending from the anterior chest to the epigastric region, with tenderness on palpation. The abdomen was soft and flat, with normal bowel sounds, and no lower extremity edema was noted. ECG showed no significant abnormalities (Figure 5), and cardiac markers remained within normal limits; however, alkaline phosphatase (ALP) and γ-GTP were slightly elevated. White C-reactive protein (CRP) was elevated (Table 3).

**Table 3:** Laboratory findings in Case 3 CRP: C-reactive protien; AST: aspartate aminotransferase; ALT: alanine transaminase; T-bil: total bilirubin; γ‐GTP: gamma-glutamyl transferase; ALP: alkaline phosphatase; CK: creatine kinase; Tn-1: troponin I

Parameters	Patient Values	Units	Reference Ranges
WBC	9900	/μL	3500-8500
CRP	7.95	mg/dl	0-0.29
AST	39	IU/L	7-38
ALT	34	IU/L	4-36
T-bil	0.61	mg/dl	0.2-1.2
γ‐GTP	67	IU/L	9-40
ALP	139	IU/L	38-113
CK	86	IU/L	60-290
Tn-I	10	pg/ml	0-26.2

**Figure 6:**
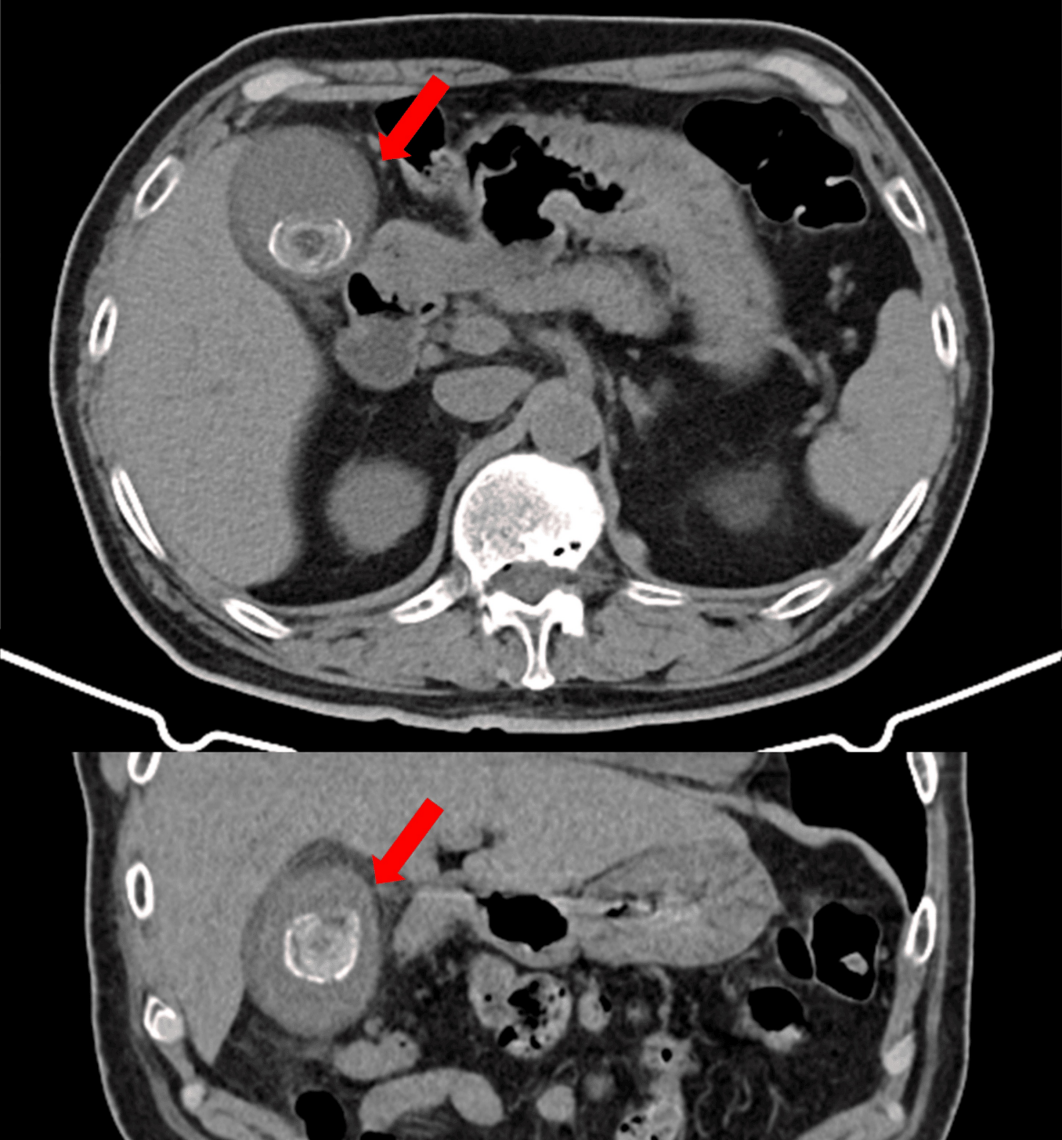
CT findings in Case 3 Chest-abdominal computed tomography showing gallstones with gallbladder enlargement and wall thickening, consistent with acute cholecystitis (red arrow).

Because ACS and aortic dissection were initially considered, a contrast-enhanced chest-abdominal CT scan was performed. Because acute cholecystitis was not suspected at first, an ultrasound was not performed. A CT revealed gallstones and gallbladder enlargement with wall thickening (Figure 6).

Although serial measurements and cardiology consultation were not performed, the Thrombolysis in Myocardial Infarction (TIMI) risk score was calculated as 2 (age ≥65 years and two coronary risk factors: hypertension and dyslipidemia), indicating intermediate risk. A repeat physical examination confirmed a positive Murphy’s sign. According to the TG18 diagnostic criteria, the patient fulfilled the requirements for acute cholecystitis, showing local signs (positive Murphy’s sign), systemic inflammation, and imaging findings. Although the TIMI risk score was 2, indicating an intermediate risk, acute cholecystitis was considered the most likely cause of the chest pain. With a Charlson Comorbidity Index (CCI) of 4 and an American Society of Anesthesiologists physical status (ASA-PS) of 2, the patient was deemed fit for surgery, and a laparoscopic cholecystectomy was performed on the same day. Intravenous cefmetazole (CMZ) was administered from the day of admission until hospital day 4, and the patient was discharged ambulatory on day 5.

The Tokyo CCU Network

The Tokyo CCU Network [7] is a regional emergency transport system in metropolitan Tokyo that coordinates prehospital triage and direct transfer of patients with suspected ACS to specialized hospitals capable of providing 24-hour cardiology care and emergent PCI.

## Discussion

Three cases were presented in this report of three patients who visited the ED with chest or epigastric pain and were initially suspected of having ACS. In all three cases, the patients were transported to or referred to the ED with chest pain and suspected ACS. However, each patient was ultimately diagnosed with a biliary condition, either acute cholecystitis or cholangitis, based on imaging and clinical findings. All patients received guideline-directed therapy: intravenous antibiotics in all cases, ERCP in one case, and laparoscopic cholecystectomy in two cases. The symptoms resolved following these interventions.

Chest pain is a frequent and potentially life-threatening presentation. It is appropriate and common for emergency physicians and prehospital providers to prioritize ruling out cardiac causes, particularly ACS, given its high mortality rate if left untreated. Prompt identification and intervention for ACS can directly affect the prognosis, justifying the initial diagnostic focus [2,3]. However, once ACS is reasonably excluded, failure to broaden the differential diagnosis may delay accurate diagnosis and treatment of noncardiac causes. Gastrointestinal disorders, particularly gastroesophageal reflux, are well-known causes of noncardiac chest pain. Other gastrointestinal etiologies, such as biliary disease, can also have atypical presentation and are more easily overlooked [1,3,8]. Although rare, acute cholecystitis can mimic ACS. In a cardiology cohort of 5,552 hospitalized patients in Japan, Ozeki et al. reported five cases of acute cholecystitis initially misdiagnosed as cardiac emergencies (<0.1%), some of which showed transient ECG changes or elevated cardiac biomarkers mimicking ischemia [4]. These findings suggest that while uncommon, biliary diseases should be considered among the noncardiac causes when chest pain persists after ACS is excluded. Chest pain caused by biliary diseases has long been known. Cope described gallstone disease as a noncardiac cause of pain mimicking that of a cardiac origin [9]. Since then, cases of cholecystitis or cholangitis masquerading as ACS have been reported [10-12].

Biliary diseases such as cholecystitis and cholangitis can present with chest pain through three primary neural pathways. First, nociceptive signals from the gallbladder and bile ducts travel via sympathetic afferent fibers to the thoracic spinal cord (mainly T4-T6), where they converge with somatic sensory neurons in the dorsal horn. This convergence can lead to referred pain perceived in the anterior chest, mimicking cardiac pain [13]. Second, afferent signals transmitted through the vagus nerve reach the medullary solitary nucleus, where excessive visceral stimulation may trigger vagal reflexes resulting in chest pain mediated by autonomic mechanisms [14]. Third, the phrenic nerve, which provides sensory innervation to parts of the gallbladder, can transmit inflammatory signals to the cervical spinal cord (C3-C5), causing referred pain to the shoulder and upper chest [15]. These mechanisms underscore the complex neuroanatomical basis for noncardiac chest pain in biliary disease.

Cognitive biases are systematic errors in judgment arising from heuristic thinking. Among these, framing bias and anchoring bias are two particularly influential forms that distort diagnostic reasoning. Framing bias refers to the tendency for contextual cues, such as referral labels, transfer designations, or prehospital categorizations, to shape the clinician’s initial diagnostic orientation before direct patient assessment takes place [16]. Anchoring bias is the tendency to adhere too strongly to an initial impression or hypothesis and to insufficiently adjust one’s thinking when new or contradictory data become available [17]. These biases often operate sequentially: framing determines the diagnostic entry point, and anchoring subsequently “locks in” that trajectory.

The high frequency of cognitive errors in the ED stems fundamentally from the characteristics of the environment and the cognitive mechanisms employed by physicians during decision-making. The ED functions as a fast-paced clinical setting, requiring emergency physicians (EP) to make clinical decisions multiple times daily. In this context, the default cognitive pathway relies on heuristics, which are cognitive shortcuts or maxims that save time and effort. Heuristics are especially useful in the ED as they ease the cognitive load and facilitate efficient throughput, but this rapid processing system is more prone to error if patient presentation is complex, evolving, or uncommon. Furthermore, frequent multitasking and task switching can lead to errors, and cognitive function can suffer when physicians experience high levels of stress and fatigue. Even greater experience does not fully mitigate risk; more experienced physicians tend to commit to a diagnosis earlier, predisposing them to premature closure and incurring an increased risk of being overconfident in an incorrect diagnosis [18].

Cardiovascular disease, a major potential cause of chest pain, was identified as the third most common initial diagnosis (10.9%) leading to diagnostic errors in the emergency setting, underscoring its role as a high-risk disease group within the emergency room. A more detailed analysis further revealed that the corresponding final diagnosis for error cases initially categorized as cardiovascular disease was “other cardiovascular diseases” in 66.7% of instances [19]. This finding aligns with the overall study conclusion that diagnostic errors frequently arise from overlooking another disease within the same organ or a disease in a closely related organ. In all three of our cases, framing bias shaped the early diagnostic trajectory. In Case 1, the patient initially arrived with the label “suspected ACS,” establishing a strong cardiac frame. At the second presentation, he was transported again through the Tokyo CCU Network under a presumed cardiac indication, reinforcing the same frame before evaluation began. Although both cardiac evaluations were negative, this cardiac contextualization contributed to the delayed recognition of rising biliary enzymes. In Case 2, transport through the Tokyo CCU Network similarly created a prehospital cardiac frame. The patient’s recent STEMI amplified the contextual pull toward a cardiac interpretation, leading clinicians to view recurrent chest and epigastric pain as potentially ischemic despite the presence of Murphy’s sign and imaging findings consistent with acute cholecystitis. In Case 3, a referral note from a primary clinic citing “possible angina” positioned the case within a cardiac diagnostic context from the outset, delaying integration of abdominal tenderness and mildly elevated biliary markers. Anchoring bias then reinforced these early frames. In Case 1, clinicians remained focused on cardiac disease during the second visit, despite repeated negative cardiac testing and evolving biliary enzyme abnormalities that warranted diagnostic reconsideration. In Case 2, the recent STEMI served as a powerful internal anchor. Minor ECG abnormalities were overinterpreted, whereas abdominal findings were underinterpreted. The fact that cardiac biomarkers were normal did not trigger a reframing of the diagnostic approach. Availability bias, where recent memorable events increase the perceived likelihood of a diagnosis [20], likely further strengthened the cardiac anchor in this patient. In Case 3, an intermediate TIMI score and early cardiac framing reinforced anchoring on a cardiac etiology, resulting in delayed recognition of biliary pathology despite compatible abdominal findings.

Together, these cases illustrate a pattern in which external framing set a cardiac trajectory before the patient encounter, and anchoring subsequently constrained diagnostic flexibility even as contradictory findings emerged [16-19]. Availability effects reinforced this chain in Case 2.

System-level features of ED workflows may have further contributed to these delays. Chest pain algorithms appropriately emphasize rapid exclusion of ACS, leading to early cardiac testing and algorithm-driven decision-making. However, this structure may unintentionally defer abdominal assessment, review of biliary enzymes, or consideration of noncardiac etiologies. Transfers through the Tokyo CCU Network and referrals for “suspected angina” naturally strengthen cardiac framing even before the clinician’s first contact. While these pathways are clinically appropriate, they highlight the importance of intentional diagnostic reassessment once ACS has been reasonably excluded.

Beyond recognizing how anchoring, availability, and framing biases contributed to diagnostic delays in these cases, it is equally important to consider strategies that may mitigate these cognitive pitfalls in future practice. Several approaches have been proposed in the literature, spanning metacognition, structured diagnostic tools, and educational interventions. Croskerry emphasized that cognitive errors are among the most frequent contributors to diagnostic failure and advocated “cognitive debiasing,” which incorporates deliberate metacognition and cognitive-forcing strategies to challenge one’s initial impressions [5]. Because many diagnostic errors stem from clinicians’ cognitive biases, several authors have proposed applying the concept of checklists, an approach that has successfully reduced errors in other high-risk, high-reliability professions such as airline piloting and nuclear plant operations, to the diagnostic process.

General diagnostic checklists are designed to help clinicians optimize their cognitive approach by offering a reproducible structure for evaluation. These lists include key steps such as obtaining one’s own complete medical history, performing a focused and purposeful physical examination, and ensuring an appropriate pathway for follow-up. Importantly, they function as cognitive forcing tools by compelling a deliberate “diagnostic time-out” (pause to reflect), thereby promoting analytic reasoning and reducing the likelihood of premature closure [21]. The differential-diagnosis checklist is designed with a single purpose: to help clinicians avoid the final common pathway of most diagnostic errors-failure to consider the correct diagnosis as a possibility. This checklist prompts clinicians to review a comprehensive list of potential causes for broadly presenting complaints (e.g., chest pain, fatigue, cough, dizziness), including “don’t-miss” diagnoses and conditions that are frequently overlooked. It is specifically intended to counteract cognitive biases by encouraging deliberate consideration of alternative explanations beyond the initially favored diagnosis. In doing so, it helps clinicians resist anchoring-fixation on the first impression and availability bias, in which easily recalled diagnoses are overestimated [21].

The use of general cognitive checklists and differential-diagnosis checklists could have mitigated the cognitive biases observed in these cases and allowed earlier recognition of noncardiac causes once ACS had been excluded.

Limitations

This study has several limitations. First, because it is based on a small case series, the frequency or clinical characteristics of biliary-induced chest pain cannot be generalized. Second, the analysis of cognitive biases was retrospective and interpretive, relying on the authors’ review of clinical documentation and case progression; thus, the presence or degree of bias could not be objectively verified. As a case-based report, it does not aim to establish causality but rather to raise awareness of how atypical presentations and cognitive pitfalls may influence diagnostic accuracy in emergency care. These inherent limitations should be considered when interpreting the findings.

## Conclusions

In patients presenting with chest pain, it is important to systematically reassess noncardiac causes once ACS has been excluded. Clinicians should remain aware that cognitive biases may contribute to diagnostic delays or errors. These cases highlight the need to maintain reflective clinical reasoning, even after life-threatening conditions have been ruled out.
